# Role of Urological Botulinum Toxin-A Injection for Overactive Bladder and Voiding Dysfunction in Patients with Parkinson’s Disease or Post-Stroke

**DOI:** 10.3390/toxins15020166

**Published:** 2023-02-17

**Authors:** Ju-Chuan Hu, Lin-Nei Hsu, Wei-Chia Lee, Yao-Chi Chuang, Hung-Jen Wang

**Affiliations:** 1 Division of Urology, Department of Surgery, Taichung Veterans General Hospital, Taichung 407, Taiwan; 2 Department of Urology, An Nan Hospital, China Medical University, Tainan City 833, Taiwan; 3 Division of Urology, Kaohsiung Chang Gung Memorial Hospital, Chang Gung University College of Medicine, Kaohsiung 807, Taiwan; 4 Center for Shock Wave Medicine and Tissue Engineering, Kaohsiung Chang Gung Memorial Hospital, Kaohsiung 807, Taiwan

**Keywords:** botulinum toxin A, incontinence, Parkinson’s disease, stroke, dyssynergia

## Abstract

Botulinum toxin A (BoNT-A) paralyzes muscle by blocking acetylcholine release at the synaptic junction. BoNT-A has shown its therapeutic effects in neurological disorders such as Parkinson’s disease (PD) and post-stroke spasticity. A high proportion of patients with PD and post-stroke develop neurogenic detrusor overactivity (nDO) and then develop urinary incontinence and overactive bladder (OAB) symptoms. This study aimed to disclose the safety and efficacy of BoNT-A injection in treating bladder and voiding dysfunction in PD and post-stroke patients by reviewing the current evidence. At present, intradetrusor injection of BoNT-A is a Food and Drug Administration (FDA)-approved third-line therapy for nDO and idiopathic OAB. Although intradetrusor injection of onaBoNT-A 200 U is already approved for nDO treatment, most researchers would like to manage PD and post-stroke patients by using onaBoNT-A 100 U intradetrusor injection to achieve long-term efficacy and reduce adverse effects. However, in contrast to its inclusion in the International Continence Society guidelines for PD treatment, the clinical use of BoNT-A for post-stroke patients is limited to experimental use due to the development of urinary retention in about one-fifth of patients. For treating urethral pseudodyssynergia, half of patients may respond to onaBoNT-A 100 U urethral injection. However, refinement is needed to reduce unwanted urinary incontinence.

## 1. Introduction

Botulinum toxin A (BoNT-A) is a neurotoxin derived from *Clostridium botulinum*. Its mechanism of action in treating overactive bladder (OAB) and urinary urgency incontinence (UUI) involves inducing flaccid paralysis via blockade of the acetylcholine release at the synaptic junction [1]. In addition, BoNT-A inhibits bladder afferent nerve firing and provides anti-inflammatory effects to manage bladder disorders. The United States Food and Drug Administration (FDA) first approved BoNT-A in 2011 for the treatment of neurogenic detrusor overactivity (nDO) and then approved it later in 2013 for refractory OAB. The expectation for intradetrusor injection of BoNT-A for reducing urinary tract dysfunction (LUTD) would be to decrease detrusor contractility, reduce bladder hypersensitivity, and eliminate painful sensations [1]. Therefore, researchers investigated the application of BoNT-A to manage the detrusor hyperreflexia (i.e., nDO) and UUI, which both originated from upper motor neuron syndrome, as seen in patients with Parkinson’s disease (PD), post-stroke, and early dementia [2].

Chronic brain disorders such as PD and stroke lead to a high proportion of LUTD in the affected patients [3]. The majority of these patients may have nDO in the cystometrography and experience UUI in their daily activity [4,5]. The occurrence of urinary incontinence (UI) in such patients may result from either nDO or impaired cognition and immobilization. Proper evaluation of micturition dysfunction in these patients is dependent on urodynamic diagnosis combined with imaging and pressure flow studies. Some patients may have concurrent detrusor underactivity, bladder neck dysfunction, and pseudodyssynergia (delay in striated sphincter relaxation or unrelaxing) [2,4,5]. Effective management of LUTD in these patients may benefit psychosocial and health-related quality of life and decrease social isolation, anxiety, depression, and fall risk.

Usually, intradetrusor injection of BoNT-A for OAB is classified as the third-line option among all OAB therapies [6]. Meanwhile, first-line management includes behavior therapies and lifestyle modifications followed by pharmacotherapy as second-line therapy [7]. Nevertheless, mainstream pharmacotherapy of OAB, which includes antimuscarinics and β3 agonists, is often problematic in older adults [6]. Constipation, dry mouth, blurred vision, and cognitive impairment are common adverse effects of antimuscarinics. Furthermore, researchers have reported a strong association between the use of antimuscarinics and risk of incident dementia [6]. Hence, of particular concern in elderly PD or post-stroke patients, procedural interventions (i.e., third-line therapies) must be optimized in older patients with motor symptoms, such as using BoNT-A injection, sacral neuromodulation (SNM), and percutaneous tibial nerve stimulation (PTNS).

## 2. Brain–Bladder Circuit

**Figure 1:**
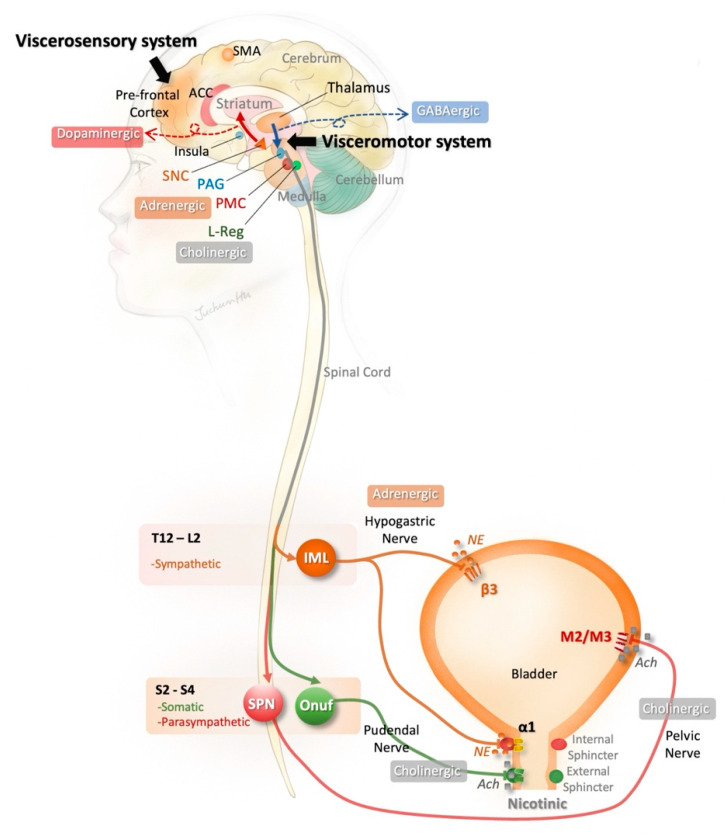
**Brain–bladder circuit for micturition.** The voiding switch is operated by the “viscerosensory system” and “visceromotor system.” The viscerosensory system consists of the prefrontal lobe, thalamus, insula, SMA, ACC, and other associated regions, and helps to manage afferent signals from the lower urinary tract. The visceromotor system includes the hypothalamus and PAG in the midbrain and mediates the efferent signals from PMC to the spinal cord. The lower urinary tract, including the urinary bladder and its outlet, are controlled by the coordination of three neural systems. The sympathetic system controls the detrusor (via β3 receptor) and bladder neck (via α1 receptor). The somatic system works simultaneously with the sympathetic system and contributes to the urethral closure through the cholinergic pathway. Activating the parasympathetic motor system results in detrusor contraction via M_2_ and M_3_ receptors. Lesions at the brain or brainstem, as in stroke and Parkinson’s disease, interfere with the brain–bladder neural circuity, resulting in lower urinary tract dysfunction. **Abbreviation: ACC**: anterior cingulate cortex; **Ach**, acetylcholine; **α1**, alpha-1 adrenergic receptor; **β3**, beta-3 adrenergic receptor; **GABA**, γ-aminobutyric acid; **IML**, intermediolateral cell column; **L**, lumbar; **L-reg**, L region (Pontine storage center); **M2/M3**, muscarinic acetylcholine receptor 2 and 3; **NE**, norepinephrine; **Onuf**, Onuf’s nucleus; **PAG**, periaqueductal grey; **PMC**, pontine micturition center; **S**, sacral; **SMA**, supplementary motor area; **SNC**, substantia nigra pars compacta; **SPN**, sacral parasympathetic nucleus; **T**, thoracic.

The lower urinary tract consists of the bladder and urethra, which are regulated by three micturition centers, including the sacral spinal center, subconscious structures (e.g., cerebellum, striate nucleus, and hypothalamus), and conscious structures (e.g., limbic cortex, frontal ascending, and parietal ascending circumduction) [4,5]. The brain–bladder circuit is illustrated in Figure 1.

Generally speaking, “urinary urgency” may be initiated by the “afferent noise” from an unstable bladder, including firing pain C-fibers, stretching Aδ/C afferents, chemical stimulating urothelium, and microcontraction of the detrusor. The afferent signals may pass through the spinal cord and medulla to the cortex [8]. The forebrain influences voluntary control of the human micturition switch and maintenance of incontinence. The prefrontal cortex receives input from the bladder pertaining to the “viscerosensory system” at the orbital prefrontal network, whereas the medial network serves as a “visceromotor system” that relays major cortical output to the hypothalamus and periaqueduct gray in the midbrain [5]. Then, the arc of the spinal cord/periaqueduct gray/pontine micturition center plays a suppressive role in activity of the urethral sphincter and bladder detrusor to maintain bladder control [5,8].

In the pathological conditions of upper motor neuron syndrome, nDO is a major cause of UUI. In lesions above the brain stem, the reflex arc of micturition is intact, whereas the existence of DO may exaggerate the micturition reflex [3,4,5]. For instance, the lesions in the basal ganglia (i.e., striatum, globus pallidus, substantia nigra, and subthalamic nucleus) play an important role in the development of neurogenic OAB [5,9]. The net effect of basal ganglia is inhibitory. The abnormal activation of putamen in functional neuroimaging has been reported in PD patients with DO [10], as well as hypersensitive bladder in ketamine-induced cystitis in a rat model [11].

## 3. Bladder Dysfunction in PD Patients

The prevalence of PD is around 0.1–0.2% in the population at any given time [12]. PD is a chronic, progressive, neurodegenerative disease characterized by the manifestation of motor symptoms such as bradykinesia, static tremor, and rigidity. These symptoms are caused by a loss of dopaminergic neurons in the substantia nigra. In addition, autonomic dysfunction is a classic non-motor phenotype of PD, including gastrointestinal malfunction, cardiovascular dysregulation, urination disturbances, sexual dysfunction, thermoregulatory aberrance, and tear abnormalities [13]. LUTD, which may present with storage and emptying symptoms, is a common non-motor sequela of PD [14] and has been reported to occur in 27–85% of PD patients during any stage of the disease [15]. In a recent meta-analysis, Li et al. [16] reported that the most prevalent storage symptom in PD patients is nocturia (59%), followed by frequency (52%), urgency (46%), and UUI (32%). One-third of PD patients may experience OAB. The emptying symptoms of PD patients may manifest as voiding difficulty, presenting symptoms of hesitancy, poor stream, and straining [15,16]. LUTS symptoms often occur five years after the onset of Parkinsonian motor symptoms [4]. Together these symptoms have substantial negative effects on patients’ quality of life and are a major cause of hospitalization and dependence upon caregivers.

Most bladder disorders in PD patients are caused by PD itself [17,18] and the occurrence of LUTS is associated with progression of motor symptoms and cognitive dysfunction [18,19]. OAB is the major issue of bladder dysfunction in patients with PD [4]. Common diseases that may cause bladder dysfunction, including urological cancer, stone disease, and urinary tract infections, must be ruled out. Questionnaires (e.g., OAB symptom score and American Urological Association Symptom Index), bladder diary, uroflowmetry, and post-void residual estimate are especially useful for the initial evaluation of bladder function in patients with PD. Because the development of LUTS in PD patients may relate to nigrostriatal dopaminergic degeneration [17,18], the first line of LUTS treatment for PD patients is to provide levodopa or other dopaminergic drugs [9]. Although not addressing LUTS directly, treatment with levodopa has been shown to improve the storage symptoms in PD patients. The second line therapies may include antimuscarinic agents and β3 agonists (i.e., mirabegron). However, the adverse effects (e.g., constipation and cognitive impairment) of antimuscarinics remain of concern, particularly for oxybutynin [4,6,9]. In addition, desmopressin (an analogue of arginine vasopressin) for nocturnal polyuria and tamsulosin (an α1-blocker) for symptoms of bladder outlet obstruction are suggested for symptom relief in PD patients [4,9,14].

For some patients with PD who do not respond well to the initial treatment of LUTS, urodynamic studies are required to differentiate the voiding dysfunction in detail, particularly using pressure-flow and video-urodynamic studies [4,9,14]. For instance, the disease of multiple system atrophy (MSA) is also a progressive neurodegenerative disorder with glial cytoplasmic inclusion, which is possibly involved in cytoskeletal alterations and neuronal degeneration [4]. Patients with MSA may have Parkinson-like motor symptoms and similar symptoms of LUTS. However, those with MSA generally show little response to the dopamine medications used to treat PD [4]. In a urodynamic investigation, Shin et al. [20] reported that DO and associated UUI were dominant in PD patients. In contrast, MSA patients may have a lower maximal flow rate, decreased compliance, detrusor underactivity, and an increase in post-void residual urine. In addition, Vurture et al. [21] suggested that nDO is almost universal in PD patients complaining of OAB symptoms. However, bladder outlet obstruction, detrusor underactivity and increased post-void residual urine may also be observed in PD patients [21,22]. Following proper evaluation of bladder dysfunction, third-line therapies can be applied to patients who discontinued pharmacotherapy or in whom drug therapy has inadequate efficacy. However, the use of SNM and PTNS may have some inherent limitations in PD patients, such as the more invasive procedure of SNM or the need for frequent visits for receiving PTNS. Therefore, intradetrusor injection of BoNT-A may offer the advantages of long-term efficacy, appropriate cost, and a less invasive procedure.

## 4. UI in Post-Stroke Patients

With the increasing growth and aging of populations, it is expected that stroke events, their long-term sequelae, and the corresponding costs will increase dramatically [23,24]. Stroke is a leading cause of adult disability. Increases are estimated to be 1.1 million and 2 million new cases annually in Europe [23] and China [24]. Using stroke survivors as an indication of prevalent stroke cases, Wang et al. [25] reported that 1.5% of adult residents of China experienced stroke. Among stroke types, ischemic stroke constituted 77.8%, intracerebral hemorrhage 15.8%, and subarachnoid hemorrhage 4.4%. A large spectrum of post-stroke LUTS is also documented, varying from UI to urinary retention [26]. In the immediate post-stroke phase, device-based management of incontinence, such as indwelled catheters or urinary pads, is most common. UI affects around half of stroke survivors in the acute phase. After the acute period, OAB symptoms are the dominant LUTS symptoms in post-stroke patients. Akkoç et al. [27] reported that two-thirds of post-stroke patients presented with urgency at six-months follow-up. In post-stroke patients, UUI causes embarrassment and distress, adding to the disability and helplessness caused by neurological deficits. Therefore, clinicians need to develop formal plans to guide UI practice, and subsequently use individualized management strategies to improve patients’ outcomes.

The exact mechanism of UI after a stroke is still unclear, but it may be a sign of patients’ poor prognosis in later life. Researchers suggest that UI presenting at 30 days after a stroke may increase the risk of one-year mortality of continent stroke survivors by four times [28]. Post-stroke UI was also associated with a negative functional outcome [29]. Hemorrhage stroke, chronic cough, aphasia, cognitive impairments, upper limb dysfunction, and fecal impaction were predictors of post-stroke UI [30,31]. However, the lesion sites of stroke seem not to correlate with patients’ urodynamic presentations [32]. At one month after stroke, Pizzi et al. [33] reported that 30% of patients with UI may present with normal functional bladder, and 48% will present with nDO; DO with impaired contractility is reported in 6%, and detrusor underactivity in 6%. Nevertheless, nDO is the most prevalent urodynamic finding and the major cause of UUI in post-stroke patients. In addition, around 10% of stroke patients may have sphincter pseudodyssynergia after stroke [5]. Therefore, urodynamic studies are necessary in the management of difficult cases of post-stroke UUI.

After the recovery period, post-stroke patients may still exhibit bladder dysfunction and UUI. Behavior therapies are the first-line treatment of such patients with OAB, including bladder training and fluid management [5]. Second-line therapies include antimuscarinics and β3 agonists. Intradetrusor BoNT-A injection may be the choice for third-line therapy for more difficult patients in order to avoid the adverse effects of medication, as well as to reach long-term efficacy and improve patients’ quality of life.

## 5. Application of BoNT-A in LUTD

The use of BoNT-A for LUTD was first described by Dykstra et al. [34] in 1988 for the treatment of patients with detrusor external sphincter dyssynergia. Following the successful demonstration of BoNT-A efficacy and safety, clinical trials were conducted for treating voiding dysfunction, especially for neurogenic DO in spinal cord injury and multiple sclerosis [2]. Generally, BoNT is classified into seven distinct neurotoxins (i.e., types A-G) that inhibit acetylcholine release at the presynaptic cholinergic neuromuscular junction to paralyze muscles [35]. Although little to no evidence supported the effects of other types, clinicians concentrated on the use of BoNT-A, including onaBoNT-A (Botox^®^, Allergan, Westport County Mayo, Ireland) and aboBoNT-A (Dysport^®^, Ipsen Ltd., Boulogne-Billancourt, France) to manage LUTD. On the other hand, serving as a powerful muscle relaxant, BoNT-A is widely used in treating the sequelae of Parkinsonism [36] and post-stroke muscle spasticity [37]. Particularly, the use of BoNT-A in urological dysfunctions of patients suffering from PD has been summarized [38].

### 5.1. Structure and Function of BoNT-A

**Figure 2:**
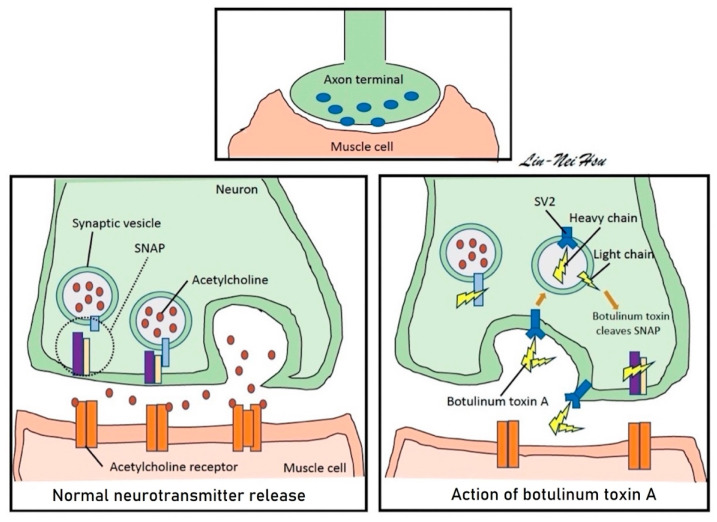
**Simplified mechanisms of BoNT-A to paralyze the detrusor muscle.** In physiology, synaptic vesicles interact with SNAP to release acetylcholine at the nerve terminals. However, via binding with SV2, the BoNT-A is endocytosed to cleave the SNAP-25 by its light chain and prevent the release of acetylcholine. **Abbreviation: BoNT-A,** botulinum toxin A; **SNAP**, synaptosomal-associated protein; **SV2**, synaptic vesicle associated protein-2.

BoNT-A is a synthesized inactive protein with a 50 kDa light chain and a 100 kDa heavy chain linked by disulfide and noncovalent bonds [39]. The major cell surface receptor of BoNT-A is synaptic vesicle protein-2 (SV2). The heavy chain binds to SV2 on the surface of the nerve ending. BoNT-A is cleaved to leave the light chain as its true active moiety due to endocytic internalization of the toxin within the nerve terminal. Then, the light chain of BoNT-A cleaves synaptosome-associated protein 25 (SNAP-25), a protein essential to the binding of synaptic vesicles to the cell membrane, to prevent neurotransmitter-containing vesicles’ exocytosis at the nerve terminal (Figure 2). SV2-immunoreactive and SNAP-25-immuoreactive nerve fibers may be distributed within the suburothelium and muscle layer in the human bladder [39].

### 5.2. Biological Effects

BoNT-A may have motor effects, sensory effects, and anti-inflammatory effects that will improve LUTD by inducing chemical denervation. BoNT-A can temporarily inactivate cholinergic transmission at the neuromuscular junction in both bladder detrusor and sphincter muscle [40]. In the bladder, BoNT-A may play a complex role in micturition reflex. For the sympathetic system, BoNT-A can inhibit the release of vesicular adrenaline and inactivate the α-adrenoceptors and β3-adrenoceptors, and theoretically facilitate the excretion of urine. In fact, BoNT-A injection mainly inhibits the parasympathetic system of the bladder by inactivation of the M2 and M3 muscarinic receptors and subsequently ameliorates the urinary storage [39]. The intradetrusor injection of BoNT-A has analgesic properties through retrograde axonal transport to decrease P2X_3_ and TRPV1 expression in the suburothelial C-fibers of the human bladder [41]. Moreover, BoNT-A accumulates in the urothelium layer to inhibit ATP release [42]. The duration of BoNT-A effects on sensory bladder disorders is typically 6–9 months [39]. In rat models, BoNT-A showed its ability to inhibit the release of calcitonin gene-related peptide and substance P from afferent nerve terminals, suggesting a potential role of BoNT-A as a treatment for neurogenic inflammation occurring in patients with nDO [43,44].

## 6. Urological Injection Techniques of BoNT-A

### 6.1. Dosage

Differences in dosing are shown for each condition and in brand models by different urological studies. The only FDA-approved doses for OnaBoNT-A BOTOX^®^ are 100 U for idiopathic OAB and 200 U for nDO treatments [45]. However, this has not limited ongoing research on dosage (e.g., 300 and 500 U) and effects of aboBoNT-A (Dysport^®^) or in off-labeled use. The units between BOTOX^®^ and Dysport^®^ preparations are not the same nor are they interchangeable. In general, 1 U of BOTOX^®^ is equivalent approximately to 3 U Dysport^®^ [46]. Moreover, dilution of the toxin, the amount of liquid injected, and the number of injection sites have varied between studies and in clinical use.

### 6.2. The Technique in Bladder Injection

**Figure 3:**
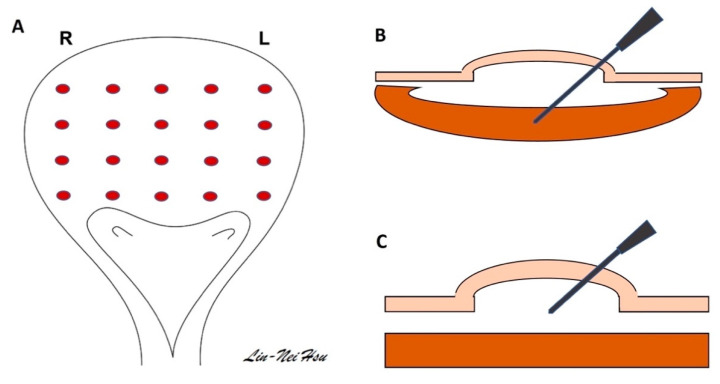
**Regular BoNT-A injection of the bladder.** (**A**) Usually, urologists mapped twenty injection sites over the bladder. (**B**) Intradetrusor method: BoNT-A solution was injected directly into the trabeculation or detrusor muscle of the bladder. (**C**) Submucosal method: Using a needle to inject and retain solution in the submucosal layer, a balloon formation was observed in the bladder after direct injection of the BoNT-A solution.

The BoNT-A solution can be injected directly into the detrusor muscle, submucosal space, and trigone [47] (Figure 3). During the injection of BoNT-A, a thin layer of bladder wall may simultaneously receive and contain the BoNT-A in the area of the detrusor and submucosal area. The trigone in the bladder base contains rich sensory fibers, which may have a role in eliciting urgency and DO. Clinically, the injection of BoNT-A into the trigone area could itself fulfill OAB treatment without inducing vesicoureteral reflux [48,49].

Usually, we prepare 100 U of BOTOX^®^ into 10 mL with dilution by normal saline. This volume is delivered to between 10 and 20 different sites of the bladder, which is typically kept at a capacity of 150–200 mL. A submucosal injection can be performed by inserting a needle into the submucosal area and observing a balloon formation in the bladder. A rigid or flexible cystoscope is able to deliver BoNT-A solution under general or local anesthesia [39,47,50].

### 6.3. The Technique in Urethral Sphincter Injection

**Figure 4:**
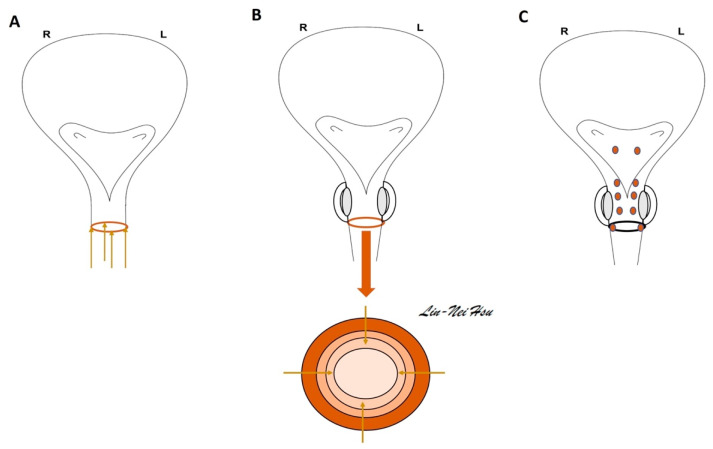
**BoNT-A injection for relaxation of the urethra.** (**A**) For female patients, it is convenient to use a 23 G 1-mL syringe and inject BoNT-A solution along the urethra at the 3, 6, 9, and 12 o’clock positions from the meatus side. (**B**) For male patients, cystoscopic injection of BoNT-A solution at the 3, 6, 9, and 12-o’clock areas of the external sphincter is common in routine urology practice. (**C**) For an extensive treatment for sphincter dysfunction, the divided dosage of BoNT-A may be injected into the trigone, superficial prostate urethra, and external sphincter for patients unresponsive to the regular injection [52].

For injection of BoNT-A into the external sphincter of the urethra, a bottle of 100 U of BOTOX^®^ is reconstituted to 4 mL with normal saline. At the 3, 6, 9, and 12 o’clock positions (Figure 4), 1 mL of BoNT-A solution in 25 U/mL was injected into the sphincter four times [51]. The cystoscope is a suitable instrument by which to perform the urethral injection in both sexes. Nevertheless, some doctors would like to inject the BoNT-A solution along the female urethra using a 23 G 1-mL syringe at the 3, 6, 9, and 12 o’clock areas of the meatus side. The BoNT-A solution can be injected in men at the circumferential sites of the urethral sphincter. For an extensive treatment of sphincter dysfunction, some may choose to inject the divided dosage of BoNT-A into the trigone, superficial prostate urethra, and external sphincter (Figure 4C) [52].

## 7. Clinical Efficacy of BoNT-A Treatment in PD

At present, intradetrusor injection using 100 U onaBoNT-A is a rational choice for treating bladder dysfunction of PD, which has proven to be a safe and effective procedure for the treatment of nDO, particularly for patients with inadequate response to antimuscarinic medications [14]. In 2009, Giannantoni et al. [53] reported four patients with PD and two patients with MSA, who received 200 U BoNT-A intradetrusor injection in 20 sites under cystoscopic guidance. The authors showed that BoNT-A injection was an effective and safe treatment for PD-related OAB symptoms and DO. Only one patient with MSA required intermittent catheterization because of an increase in post-void residual urine. In the following study by the same research team [54], 100 U BoNT-A intradetrusor injection was administered to manage eight patients with PD who were refractory to antimuscarinics. The clinical and urodynamic improvements in OAB symptoms of these PD patients lasted for at least 6 months. In 2010, Kulaksizoglu and Parman [55] reported positive results in 16 PD patients by using the flexible cystoscopic injection of 500 IU aboBoNT-A at 30 sites (trigone spared), including the improvement of urinary symptoms and incontinence and the relief of caregivers’ burden through nine months of observation.

In another comprehensive study, Anderson et al. [56] treated 20 clinic patients with PD with incontinence using 100 U onaBONT-A injection of the bladder under local anesthesia. The authors used a flexible cystoscopic instrument to disperse the solution of onaBONT-A (10 U/mL) into 10–20 submucosal/detrusor sites of the bladder, including the trigone. Results of that study showed that intradetrusor injection of BoNT-A can be safely utilized in male patients with PD who also have benign prostatic hypertrophy. Moreover, Vurture et al. [57] reported that the success rate was approximately 80% in the intradetrusor injection of onaBoNT-A 100 U for DO-driven storage symptoms of PD patients. Additionally, a repeat injection can increase the success rate to 87.5%. The rate of urinary retention requiring clean intermittent catheterization was 12.5%. In 2021, Atamian et al. [58] reported their treatment results of intradetrusor BoNT-A injection in 16 PD patients with UUI. Among these patients, 60% achieved improvement and 28% needed intermittent self-catheterization.

Urethral sphincter dysfunction may be an issue of voiding dysfunction in PD patients, including pseudodyssynergia or delay in striated sphincter relaxation [3]. Despite the rationale and success of chemical sphincterotomy in the first BoNT-A sphincter injection for detrusor sphincter dyssynergia in a patient with spinal cord injury [34], this procedure has yet to be widely utilized among patients with spinal cord injury. Regardless of whether injections are performed transperineally or transurethrally, previous studies have confirmed treatment efficacy [2]. About 50% of patients can achieve successful treatment with a decrease in urinary tract infections, nDO, and post-void residual urine [59]. However, nearly half of such cases developed UI and persistent incomplete bladder emptying, limiting the utility of urethral injection of BoNT-A [60,61]. Jiang et al. [62] reported that 100 U onaBoNT-A urethral sphincter injection is suitable to treat urethral sphincter hyperactivity, including in PD patients. The authors reported that two of three PD patients experienced satisfactory outcomes. Clearly, the BoNT-A urethral sphincter injection of PD does need some refinement, in light of the previous studies [60,61,62]. For example, a videourodynamic study may aid in the accurate diagnosis of dysfunctional voiding patterns in PD patients. Lee et al. [63] suggested that patients who were found to have a tight bladder neck during the videourodynamic study had less favorable therapeutic outcomes from BoNT-A urethral injections.

## 8. Clinical Role of BoNT-A Treatment in Post-Stroke

The ability to urinate independently is an important issue associated with human dignity [64]. Direct stroke-induced damage to the neuromicturition pathway causes involuntary leakage of urine accompanied by urgency in 40% to 60% of people admitted to hospital after a stroke [64]. First-line behavioral therapy may increase independent voiding behavior to control LUTS [64,65]. Second-line therapies of antimuscarinics and mirabegron also may improve OAB symptoms and not affect cognitive function during short-term observation [64,66]. However, post-stroke incontinence may last for a long time and not recover spontaneously. Third-line therapies may have a role in treating post-stroke incontinence. Evidence has shown that PTNS has little or no difference in the continence of participants after treatment [64]. SNM requires a surgical procedure with implantation of the InterStim^®^ device for bladder and bowel control (Medtronic, Minneapolis, MN, USA), providing continuous stimulation through close nerve contact. Despite a high success rate in treatment, SNM may have adverse effects such as pain (15–42%) and infection (3.4–6.1%) at the implant site. Moreover, the surgical revision rate of SNM may be as high as 33% [67,68].

Theoretically, BoNT-A injection in the lower urinary tract may be a third-line adjuvant therapy for post-stroke incontinence. However, only a few studies have demonstrated successful outcomes of BoNT-A treatment in these patients. In 2006, Kuo [69] reported that 12 post-stroke patients with nDO received bladder submucosal injection of 200 U onaBoNT-A, in which only 50% of participants benefited from improvement of incontinence. Another 25% of patients developed transient urinary retention in the first postoperative week. The therapeutic effect declined gradually after 3 months and symptoms relapsed at month 6. In 2014, Jiang et al. [70] reported their experience in treating post-stroke bladder dysfunction by using 100 U onaBoNT-A intradetrusor injection, in which 17.4% of post-stroke patients developed acute urinary retention. Additionally, the therapeutic duration of these post-stroke patients is similar to those of control patients with OAB. Results of that study suggest that using 100 U onaBoNT-A intradetrusor injection for post-stroke patients is a rational dosage to reach a proper therapeutic effect and to avoid adverse effects. For treating pseudodyssynergia of post-stroke patients with difficult urination, Chen and Kuo [71] applied 100 U onaBoNT-A to external sphincter injections in 12 patients, of whom, 91% resumed spontaneous voiding.

As illustrated in Table 1, sufficient evidence has shown the beneficial effects of intradetrusor injection of BoNT-A in treating the UUI of PD patients. In contrast to the guidelines of PD treatment [14], urological BoNT-A injection for post-stroke patients remains an experimental entity in clinical practice. Further selection of suitable patients and refinements of technique may help to promote the usage of BoNT-A injection in treating voiding dysfunction in post-stroke patients.

## Figures and Tables

**Table 1:** Characteristics among studies of botulinum injection in patients with CVA and PD.

Etiology	Author (Year)	Group/ Botulinum Brand	Patient Number (Male/Female)	Mean Age (SD)	Mean Duration of Disease (SD)	Injection Site	Dosage (Sites)	Anesthesia	Post-Injection Outcome			Adverse Events (Events/All Cases)
									Urodynamics	Clinical Outcome	Response Rate	
**PD**	Giannantoni (2009) [53]	Botox	4 (0/4)	76.3 (4.8)	9 years (3.5)	Detrusor (Trigone including)	200 U in saline 20 mL (20 sites)	IVGA	**IDC volume**: Significantly increased (+234 mL) at 3rd month **CMG capacity**: Significantly increased (+225.5 mL) at 3rd month **Pdet.Qmax**: Significantly reduced (−3.5 cm H_2_O) at 3rd month **Qmax**: Significant reduced (−11.3 mL/s) at 3rd month **PVR**: Significantly increased (+88.8 mL) at 3rd month	Significantly improved daytime and nighttime Frequency Completely resolved daily UI (100%) Significantly improved I-QOL(+52.5)	4/4 (100%)	0/4 (0%) UTI 3/4 (75%) Dysuria 3/4 (75%) Voiding difficulty
	Kulaksizoglu (2010) [55]	Dysport	16	67.2 (5.1)	6 years	Detrusor	500 U in saline 30 mL (30 sites)	LA	**CMG capacity**: Significantly increased (+136 mL at 3rd month,+180 mL at 6th month, +183 mL at 9th month, +76.5 mL at 12th month) **Pdet at IDC**: Reduced (−28 cm H_2_O in men, −12 cm H_2_O in women) **Persistent urodynamic UI**: None after injection	Significantly reduced SEAPI score at 3rd, 6th, 9th and 12th month 6 incontinent patients at baseline: all with reduced UUI episode	16/16 (100%)	0/20 (0%) AUR need catheterization
	Giannantoni (2011) [54]	Botox	8 (1/7)	66 (3)	NA	Detrusor (Trigone including)	100 U in saline 10 mL (10 sites)	IVGA	**Complete resolution of IDC**: 3/8 (37.5%) **CMG capacity**: Significantly increased at 1st, 3rd, and 6th month **PVR**: increased at 1st month, markedly decreased at 3rd and 6th month **Qmax**: No significant change **Pdet.Qmax**: No significant change	Significantly decreased frequency (daytime and nighttime) and UI Significantly improved I-QOL (+43) and VAS (+3.5) at 6th month	NA	2/8 (25%) AUR need catheterization 0/8 (0%) UTI
	Jiang (2014) [70]	Botox	9	73.6 (11.2)	NA	Detrusor	100 U in saline 10 mL (20 sites)	IVGA	**CMG capacity**: Increased (+17 mL at 3rd month) **Qmax**: No significant change **Pdet.Qmax**: No significant change **PVR**: Significantly increased (+77.3 mL at 3rd month)	Significantly improved USS (−1.28) Improved urgency (−13 times) Improved UUI (−1.1 times)	NA	1/9(11.1%) AUR need catheterization 3/9(33.3%) PVR > 150 mL 1/9(11.1%) Voiding difficulty need strain 1/9(11.1%) Hematuria 2/9(22.2%) UTI
	Anderson (2014) [56]	Botox	20(12/8)	70.4	10.6 years	Detrusor(Trigone including)	100 U in saline 10 mL (10–20 sites)	LA	**VV**: No significant change at 1st, 3rd, and 6th month **Qmax**: Significant reduced (−4.7 mL/s) at 1st month, but not in 3rd and 6th month **PVR**: Significantly increased at 1st month (+106 mL) and 3rd month(+40 mL), but not in 6th month	Significantly improved UUI at 1st, 3rd, and 6th month Significantly improved AUA symptom scores at 1st, 3rd, and 6th month	20/20 (100%)	0/20 (0%) AUR need catheterization 2/20 (10%) UTI 0/20 (0%) Significant hematuria
	Knüpfer (2016) [72]	Botox	10 (6/4)	67.9 (5.4)	9.2 years (8.2)	Detrusor (Trigone including)	200 U in saline 20 mL (20 sites)	NA	**CMG capacity**: Significantly increased (+136 mL) **Pdet.Max at voiding**: Significantly reduced (−40 cm H_2_O) **Compliance**: Increased (+11.1 mL/cm H_2_O) **VV**: Significantly increased (+115 mL) **PVR**: scantly increased (+16 mL) **Urodynamic DO**: Markedly reduced (90% to 20%) **Qmax**: No significant change	Significantly improved frequency, nocturia and daily pad Significantly improved ICIQ score	10/10 (100%)	0/10 (0%) AUR need catheterization 0/10 (0%) UTI 0/10 (0%) Hematuria
	Vurture (2018) [57]	Botox	24 (17/7)	77.2 (7.5)	9.8 years (5.7)	Detrusor (Trigone including)	100 U in saline 10 mL (20 sites)	LA	**PVR**: Significantly increased (+108 mL)	Significantly decreased daily pad amount and UUI	19/24 (79.2%)	3/24 (12.5%) AUR need catheterization 6/24 (25%) UTI
**CVA**	Chen (2004) [71]	Botox	11 (5/6)	66.5 (14.7)	NA	Sphincter	100 U in saline 4 mL (4 sites)	IVGA	**Pdet.Qmax**: Significantly reduced (−24 cmH_2_O) **Qmax**: Significantly increased (+3.1 mL/s)	Significantly improved IPSS and QoL index in Botox group IPSS −13.6 in Botox vs. −4 in Control QoL Index −2.4 in Botox vs. −1.2 in Control	10/11 (91%)	0/11 (0%)
		Control	10	65.4 (15.5)		None	None	None	NA	NA	4/10 (40%)	0/10 (0%)
	Kuo (2006) [69]	Botox	12 (6/6)	72.4 (5.7)	NA	Detrusor	200 U in saline 20 mL (40 sites)	IVGA	**IDC volume**: Significantly Increased at 1st month (+139.9 cm H_2_O) but not at 3rd month (+56.3 mL) **CMG capacity**: Significantly increased at 1st month (+144.9 mL) but not at 3rd month (+56.2 mL) **Pdet.Max at voiding**: Reduced (−5.4 cm H_2_O at 1st month and −7.5 Cm H_2_O at 3rd month) **PVR**: Significantly increased at 1st month (+123 mL) but not at 3rd month (+31.5 mL)	Improved incontinence grade (−1.3 at 1st month and −0.9 at 3rd month) Significantly increased grade of voiding difficulty (+1.5 at 1st month and +0.7 at 3rd month) 7/12 (58%) voiding difficulty	6/12 (50%)	3/12 (25%) AUR need catheterization 21% Mild hematuria 25% UTI
	Jiang (2014) [70]	Botox	23	73.6 (7.5)	NA	Detrusor	100 U in saline 10 mL (20 sites)	IVGA	**CMG capacity**: Significantly increased (+160 mL at 3rd month) **Qmax**: No significant change **Pdet.Qmax**: No significant change **PVR**: Significantly increased (+112.5 mL at 3rd month)	Improved USS (−0.57) Improved urgency (−8.3 times) Significantly improved UUI (−7.8 times)	NA	4/23 (17.4%) AUR need catheterization 12/23 (52.2%) PVR > 150 mL 17/23 (73.9%) Voiding difficulty need strain 2/23 (8.7%) Hematuria 1/23 (4.3%) UTI

**Abbreviation: AUA symptom score**, American Urological Association symptom score questionnaire; **AUR**, Acute urine retention; **CVA**, Cerebrovascular accident; **CMG**, cystometry; **DO**, Detrusor overactivity; **ICIQ**, International Consultation on Incontinence Questionnaire; **IDC**, Involuntary detrusor contraction; **IPSS**, International Prostate Symptom Score; **I-QoL**, Incontinence quality of life; **IVGA**, Intravenous anesthesia; **LA**, Local anesthesia; **NA**, No available data in the published paper; **PD**, Parkinson disease; **Pdet**, Detrusor pressure; **Pdet.Max**, Maximum detrusor pressure during voiding; **Pdet.Qmax**, Detrusor pressure at peak flow rate; **PVR**, Post-voiding residual volume; **Qmax**, Maximum flow rate; **QoL**, Quality of life; **SEAPI**, stress, emptying, anatomy, protection, inhibition Incontinence Quality of Life Assessment questionnaire; **UI**, Urinary incontinence; **USS**, Urgency severity score; **UTI**, Urinary tract infection; **UUI**, Urge urinary incontinence; **VAS**, Visual analogue scale; **VV**, Voiding volume.

## Data Availability

Not applicable.
